# Supplementary figures and images for: Identification of Clec4b as a novel regulator of bystander activation of auto-reactive T cells and autoimmune disease
Source: PLoS Genet. 2020 Jun 4;16(6):e1008788. doi: 10.1371/journal.pgen.1008788 (PMC7297379; doi:10.1371/journal.pgen.1008788)

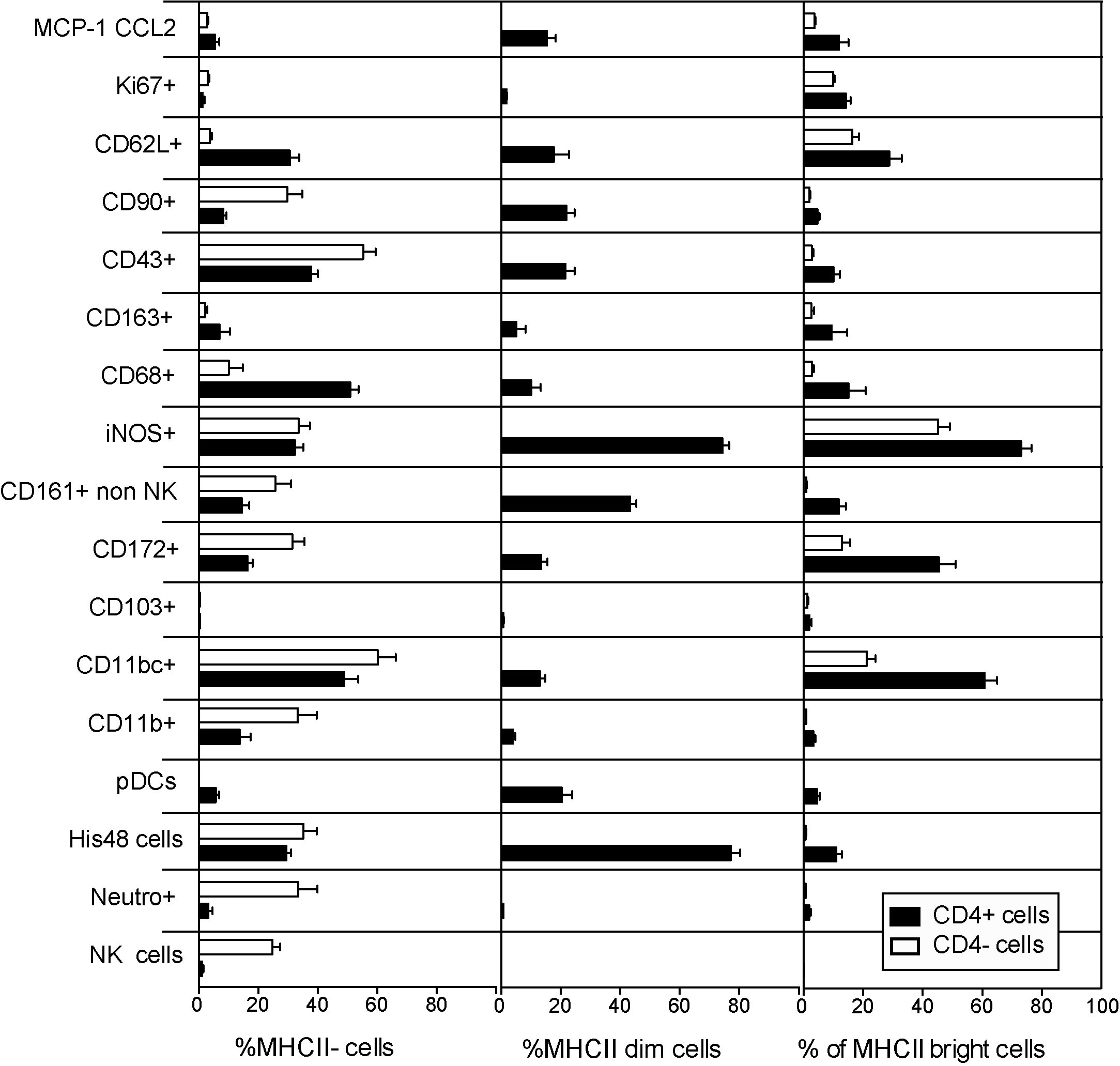

Supplement: S1 Fig — Three spleens harvested from day 3 after oil injection were selected first for T cells were the negative fraction was collected and selected a second time for CD4 expression. The twice selected cells were either CD4+ or CD4-, each represented as 3 samples. The samples were then labeled with fluorescence conjugated antibodies to identify subsets of cells. Since there appeared to be 3 clear subsets of CD4+ DCs depending on the MHCII expression the cells were first gated as either MHCII negative, MHCII high/bright and MHC dim. The value on the x axis is the present of total number of cells. (TIF) [file pgen.1008788.s001.tif]

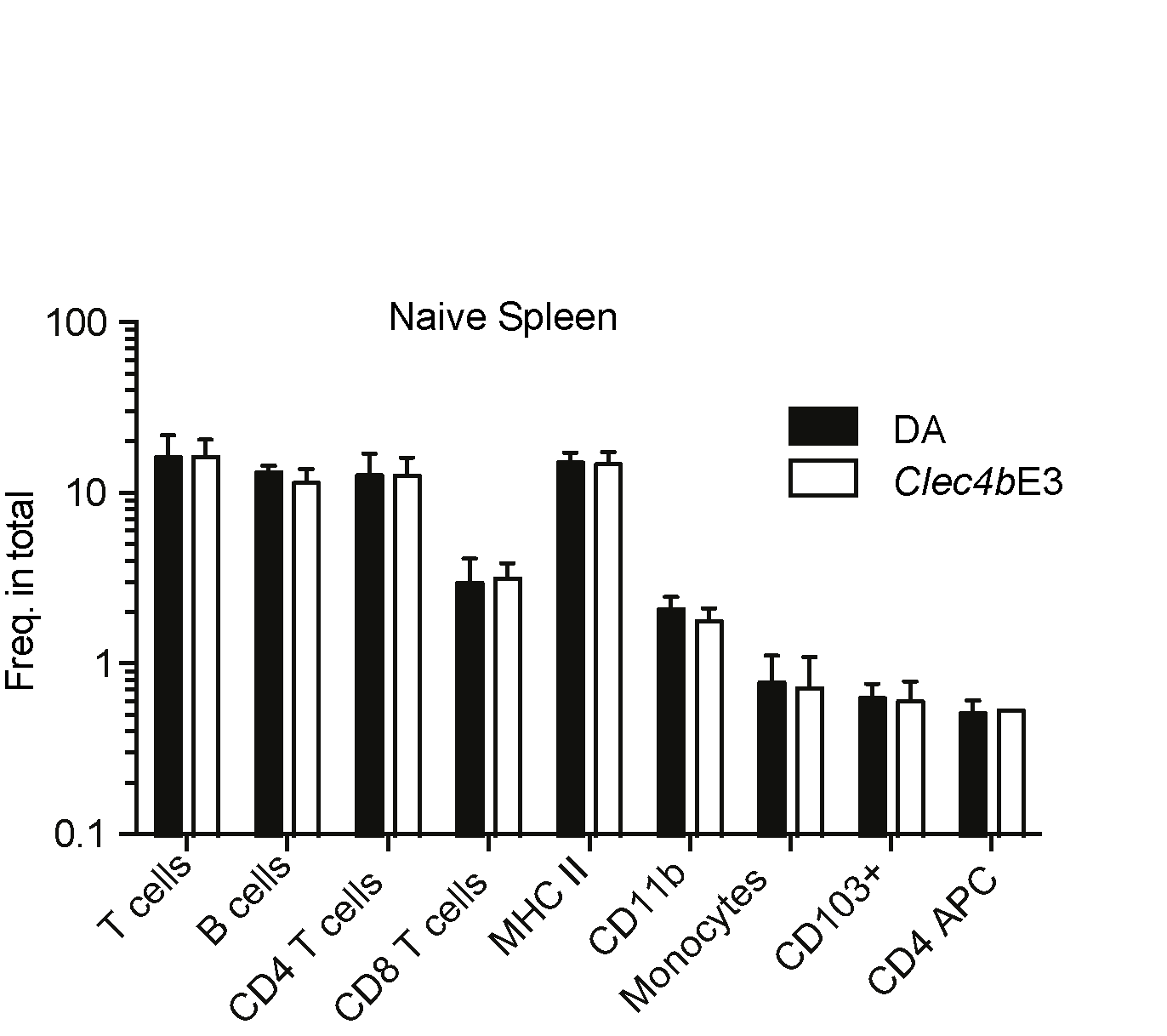

Supplement: S2 Fig — (TIF) [file pgen.1008788.s002.tif]

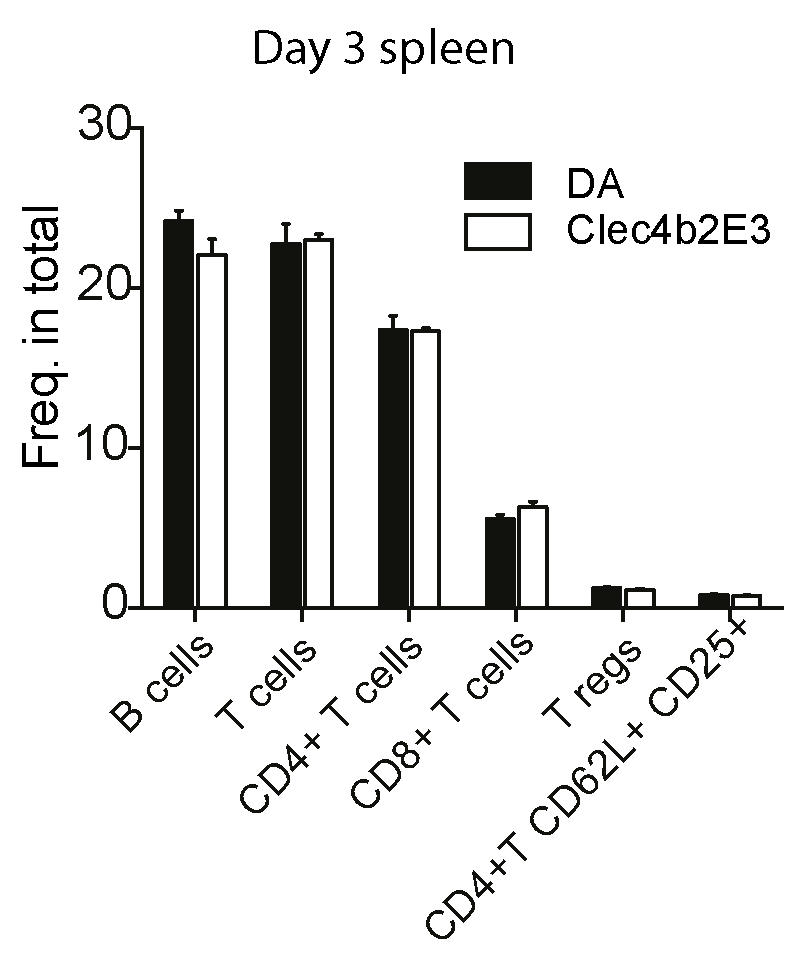

Supplement: S3 Fig — (TIF) [file pgen.1008788.s003.tif]
