# Supplementary material for: Identification of Clec4b as a novel regulator of bystander activation of auto-reactive T cells and autoimmune disease
Source: PLoS Genet. 2020 Jun 4;16(6):e1008788. doi: 10.1371/journal.pgen.1008788 (PMC7297379; doi:10.1371/journal.pgen.1008788)
Supplement: Attachment — Submitted filename: response to reviewers.docx [file pgen.1008788.s005.docx]

Reviewer's Responses to Questions

**Comments to the Authors:
Please note here if the review is uploaded as an attachment.**

Reviewer #1: Bäckdahl et al perform positional cloning and suggest a polymorphism in Clec4b confers resistance to arthritis in rats. They find that Clec4b is primarily expressed in CD4+ dendritic cells (DCs) and that mice carrying the susceptibility allele show an increased activation of CD4+ DCs upon stimulation. They then attempt to demonstrate, using both in vitro and in vivo assays, that the resistance allele in DCs suppresses T cell activation.

I have three major concerns:
1 – The authors never perform a complementation assay to prove that Clec4b is responsible for the phenotypes observed. This needs to be addressed experimentally.

*Response to 1: We have now included data using a monoclonal antibody towards Clec4b/Dcar that confirms the presence of the gene/protein on CD4+ dendritic cells in the result section 2 of the manuscript. We have shown how this DC population later deviate in activation status rapidly between the two genotypes and CD4+DCs displaying enhanced activation already day three after oil injection in the Clec4b deficient DA strain. We also show that the CD4+ DCs can inhibit T cell proliferation in cocultures with naïve T cells and also in vivo. The T cells are already arthritogenic at day 5 and can then transfer disease to naïve recipients.*

2 – Although the authors present various in vitro and in vivo assays, it is not clear that the biological effects observed are intrinsic to DCs. This can be addressed with the following series of in vitro experiments. (1) T cell responses from both strains of mice can be stimulated in vitro. This will allow to unequivocally determine whether the alleles impact T cell responses. (2) DCs from both strains should be cultured in vitro with naïve allogeneic T cells. This will allow to determine if the alleles expressed in DCs impact T cell activation.

*Response to 2.1 We are aware that there was an error in the text to result section 6 that lead to some confusion, which has now been corrected. To clarify, in the experiment, we performed a set of criss-cross experiments with CD4^+^ and CD4- DCs from both genotypes co-cultured with T cells from either DA or Clec4b.E3. We showed that CD4+DCs from Clec4b.E3 efficiently downregulated bystander activated T cells, whereas DA induced T cell proliferation despite of the T cells genetic origin.*

*Response to 2.2 A set of allogenic stimulations have been performed but no Clec4b regulation could be observed as now shown in figure 6b. Thus, as now discussed the Clec4b regulatory effect maybe not involved allogenic T cell activation.*

*By the way the animals used in the paper are rats, not mice.*

3 - The authors often mention the suppressive effect of the resistance allele on DC function. It is not clear how the assay was designed to truly monitor suppression of T cell activation as opposed to a decrease in T cell activation. A true suppression assay must be performed to claim suppressive activity.

*Response to 3. We fully agree with this comment regarding the need to distinguish suppression from decreased T cell activation. We have now tried to clarify this by more clearly pointing out that the Clec4b DC limits the autoreactive T cells to be activated and limits bystander activation. We have changed all suppression terminology to limit or downregulate.*

Minor comments:
The nomenclature for designating DC should potentially be revised in line with the recent designations. I believe that the authors are referring to a subset of cDC2, namely CD4+ cDC2. It is not clear why the authors avoid using the recent nomenclature.

*Response: CD4+ cDC2 dendritic cell has been well defined in mouse but is still poorly characterized in rat. Only one paper has mentioned cDC2 in rat why we discuss the terminology but have chosen not to use it in the manuscript.*

References are missing to support the first statements in the introduction.

*Response: We have now included additional references in the first section of the introduction.*

Figure 2 – There are white and black bars in the figure. It is not clear what these represent.

*Response: we have added more labels in the figure 2. Explanations are now hopefully more obvious.*

Why is it unexpected that the Clec4b is less expressed in the DA mice carrying the stop codon?

*Response: We thought it was unexpected as there is no reason to believe that a mutation leading to a truncated protein should be less expressed. However, we agree that “unexpected” is too strong wording and we have now rephrased it to avoid any misunderstanding.*

Figure 2b. The relative expression of Clec4b in B cells and T cells is not shown.

*Response: Relative expression in B and T cells was not included since T cells and B cells were pooled but we have included clec4b expression in B and T cells for the Clec4b antibody, now included in figure 3f.*

Figure 6. It is mentioned that the DCs suppress T cell activation. Is it truly suppression or lack of activation?

*Response: We argue that since the mutation cause a truncated protein it would not be correct to claim that the corrected null mutation in DA.Clec4bE3 could cause a lack of activation, since in the susceptible strain the protein is absent and unable to perform its function. We believe that when the receptor is expressed it corrects bystander activation, rather than inducing T cell suppression. Thus, we have rephrased this statement which is now added to the text to result in section 6.*

Figure 6. The data presented in panel D is very difficult to interpret. According to the labelling in the figure, the greatest effect seems to come from the genotype of the T cells. This contrasts with the information written in the text.

*Response: We have now reworked Figure 6, the unclear figure section 6d have been removed and replaced by the allogenic stimulation experiment and the day 3 OIA derived proliferation experiment replaced it. The inaccurate text has also been changed. Gating strategy has been included for the CD25+ proliferating T cells that are investigated through the section.*

Panel 6E is mentioned in the text, but is not present in the figure.

*Response: This error has been corrected and the figure and corresponding text have been revised and changed.*

The y axes in figure 7C appear to be non-linear. The interpretation of the data is difficult.

*Response: The figure was unfortunately with a very low resolution and out of focus and has now been updated.*

There are numerous grammatical and syntax errors throughout. In addition, the nomenclature is sometimes erroneous, not uniform, or not standard. For example, CD11bc should read CD11b/c; snps are usually abbreviated as SNPs; cells were not sorted – they were selected with magnetic beads; etc. The text and figures need to be carefully revised.

*Response: we have put a great deal of effort in to being better coherent in the text and consistent with the nomenclature and rephrasing inaccurate expressions.*

Reviewer #2:

This is an interesting study where the authors describe the possible identification of a new rat autoimmune arthritis gene and suggest a mechanistic explanation. While the findings are of potential interest there are several issues in the study that were not clear, including assumptions attributed to a gene that was not clearly studied, and therefore lacked mechanistic specificity. Here are my comments including major and minor ones:

1, Given that the locus on chromosome 4 was identified in PIA and the congenics were protected in PIA (figure 1) it was not clear why the authors used the milder OIA to fine map the region.

*Response 1: The argument for using OIA has now been clarified in the text.* *We chosed to fine map the underlying polymorphisms within the Pia7 locus for association with arthritis using OIA, which gives a milder arthritis compared with PIA. In fact, the same locus as Pia7 has also been mapped in OIA and designated Oia2 (7).*

2, More details about the breeding of the congenics should be provided as it is not clear how many generations were backcrossed and whether contaminating E3 fragments elsewhere in the genome were in fact excluded (this is critical in this study). What markers were used in the breeding?

*Response 2: This is very important information that was missing but has now been included in both the result section 1 and in materials and methods.*

3.1, The Clec region had been previously implicated in OIA by Lorentzen et al. In this study the authors tried to refine that association. Clec4b was implicated as the disease gene in the 200kb interval primarily **because E3 and BN shared the same allele,** while DA and BN had the same Clec4e allele. However, there is a co-localizing locus in a DAxF344 intercross and in congenics, and DA has the same alleles as F344 at both gene SNPs as reported in figure 1B. That raises concern about whether this is in fact the gene accounting for arthritis effect, or whether there is another gene elsewhere (E3 contamination).

3.2, **Additionally, it was surprising to see the authors exclude the intergenic and intronic SNPs at Clec4b, Clec4d and Clec4d simply because they were not in coding regions, particularly given that it is now known that those distant elements may regulate gene expression.** Therefore, it appeared to me that there was not enough evidence to implicate only the coding SNP in Clec4b and exclude the others.

*Response 3.1: The chromosome 4 locus Cia3 has been identified in DAxF344 F2 crosses however the Cia3 region is more that 100Mbp from the Pia7 locus and DA.Clec4bE3 fragment which in the DAxBN F2 cross from Griffiths et al is denoted Cia13, which the authors themselves in the manuscript claim that Cia13 is a completely segregated locus from the DAxF344 Cia3. Furthermore, our congenic strain has been back-crossed to DA for more that 19 generations which limits greatly the amount of contaminating background genes DA.Clec4bE3 may have. We have now described in detail how the different subcongenics were produced to clarify back-crosses and recombination events.*

*Response 3.2. We do agree with the comment about intragenic SNPs and the possible effects on closely situated genes. We have now included gene-expression of all APLEC encoded genes as well as the surrounding Dcir genes in Figure 1. We now showed that no other genes that Clec4b show significant differential expression.*

4.1, It was also surprising that the only “functional data” that really relates to Clec4b were mRNA levels. The authors should have shown changes in Clec4b protein levels (none is shown despite extensive flow cytometry looking at other proteins), or protein function, or protein localization in the plasma membrance, or in signaling pathways specifically regulated by Clec4b, or that knocking down Clec4b affected a phenotype. But none of that was shown and therefore the implication of Clec4b in the CD4+APC-regulated behavior appeared speculative.

4.2, How do the authors think that Clec4b in APCs would regulate autoreactive T cell development/proliferation?

*Response 4.1: This is of course a much valid point but up until June this year there has not been a monoclonal antibody to Clec4b available. Before this we made numerous attempts in trying to make a specific monoclonal antibody but did not succeed. We were however happy to get help from Daws et al and have included the CD4^+^DC staining with their antibody to Clec4b and we can now show it is indeed expressed on this population and only for the DA.Clec4b.E3 congenic*.

*Response 4.2: The following explanation to this question is now described in both the result section 6 and 7. We think that the lack of Clec4b induces a rapid bystander activation of T cells after an intradermal oil injection in the DA strain whereas this is quickly prevented in the Clec4b expressing DCs.*

5, Unclear why authors use two different nomenclatures to refer to the E3 congenics: please choose one.

*Response 5: We now only use Clec4b^E3^through out the manuscript.*

6, In addition to spleens, it would have been interesting to know whether the synovial tissue expression of the Clec genes differed between DA and congenic.

*Response 6: This experiment has been attempted previously but we were unable to get good quality of the RNA*. *We did not make more emphasis on this as the results will not change the concept, rather it will be included in forthcoming studies to study downstream mechanisms.*

7, The authors should comment on whether the Clec region has been associated with rheumatoid arthritis or other autoimmune diseases in humans.

*Response 7: We have now included data on the human DCIR association.*

8, APC and DC appear to be used interchangeably in the manuscript which was a little confusing and could be changed.

*Response 8: All APC has been changed to DC in the manuscript.*

9, Results, section 4, paragraph 2: reference figure 3c but in fact should be figure 4e.

*Response 9: Typo error corrected reference for figure 3c now 4e*.

10, Results, section 5, last paragraph: CD62L data not shown and it is not clear what the authors think of those results.

*Response 10: Data for T cells expressing is now CD62L shown and discussed in section 5.*

11, ConA has been shown to induce murine macrophage apoptosis: did the authors check for that? Is it possible that macrophages from the two strains differed in susceptibility to apoptosis and that was the difference in mediating T cell responses?

*Response 11: A new sentence has been added to result section 6 There was no difference in viability after Con A between the two strains.*

12, How is ConA affecting Clec4b expression, and how is Clec4b interfering with autoreactive T cell development and/or responsiveness?

*Response 12: To explain this we have now included the following section into the second paragraph of the discussion: “We have here identified two triggers of Clec4b; mineral oil and Con A, which will activate DCs lacking Clec4b expression, leading to uncontrolled T cell proliferation. The presence of the lectin receptor Clec4b/Dcar on the DC surface cause a more controlled activation of DC with Clec4b^E3^, compared with DC with Clec4b^DA^, inducing less expression of proinflammatory genes such as Stat1, Irf4 and iNos, that could limit bystander T cell activation.”*

13, The authors used the term ‘positional cloning’ in the title, but then refer to the SNP as ‘arthritis-associated’ in the results: please clarify.

*Response 13: This has been changed in the text, the SNP is “arthritis-regulating”.*

14, It was not clear which strain was studied in figure 3 but it would be interesting to see a comparison between both strains.

*Response 14: We have now added the following text to the legend of figure 3 “No strain comparisons for the CD4+ subsets showed any significant differences”.*

There were also several minor text typing errors.
 	*Response: We have now tried to correct these errors.*

**Have all data underlying the figures and results presented in the manuscript been provided?**
Large-scale datasets should be made available via a public repository as described in the *PLOS Genetics* [data availability policy](https://eur01.safelinks.protection.outlook.com/?url=http%3A%2F%2Fjournals.plos.org%2Fplosgenetics%2Fs%2Fdata-availability&data=02%7C01%7Crikard.holmdahl%40ki.se%7C81a810bf3db245fb4d7908d73968a844%7Cbff7eef1cf4b4f32be3da1dda043c05d%7C0%7C0%7C637040993981871200&sdata=EpHje7MI0ECbXCKd%2BZRqovXoc8gDocrFNbhFMGBms1o%3D&reserved=0), and numerical data that underlies graphs or summary statistics should be provided in spreadsheet form as supporting information.

Reviewer #1: No: The data is plotted in bar graphs showing mean and SD. It would be best if the data points were shown. Some of the representative flow cytometry profiles are missing.

Reviewer #2: No: breeding strategy and markers used were not provided.
some flow cytometry mentioned in the manuscript was also not shown.

PLOS authors have the option to publish the peer review history of their article ([what does this mean?](https://eur01.safelinks.protection.outlook.com/?url=https%3A%2F%2Fjournals.plos.org%2Fplosgenetics%2Fs%2Feditorial-and-peer-review-process%23loc-peer-review-history&data=02%7C01%7Crikard.holmdahl%40ki.se%7C81a810bf3db245fb4d7908d73968a844%7Cbff7eef1cf4b4f32be3da1dda043c05d%7C0%7C0%7C637040993981871200&sdata=KQ8T%2BeA8bWOsZQvhTQJinZV%2F0LZrpb7M9FmJe5pe%2FYo%3D&reserved=0)). If published, this will include your full peer review and any attached files.


If you choose “no”, your identity will remain anonymous but your review may still be made public.


**Do you want your identity to be public for this peer review?** For information about this choice, including consent withdrawal, please see our [Privacy Policy](https://eur01.safelinks.protection.outlook.com/?url=https%3A%2F%2Fwww.plos.org%2Fprivacy-policy&data=02%7C01%7Crikard.holmdahl%40ki.se%7C81a810bf3db245fb4d7908d73968a844%7Cbff7eef1cf4b4f32be3da1dda043c05d%7C0%7C0%7C637040993981881192&sdata=HRWgHPzEE%2BkGUyQkDR3AFMuYNrPFMGY0w6r2OWb%2F3Ts%3D&reserved=0).

Reviewer #1: No

Reviewer #2: No

**R1**

**Major comments**

**1 Make a complementation assay to prove Clec4b is responsible for the phenotypes.**
