# Supplementary material for: Identification of Clec4b as a novel regulator of bystander activation of auto-reactive T cells and autoimmune disease
Source: PLoS Genet. 2020 Jun 4;16(6):e1008788. doi: 10.1371/journal.pgen.1008788 (PMC7297379; doi:10.1371/journal.pgen.1008788)
Supplement: Attachment — Submitted filename: Response to Reviewers.docx [file pgen.1008788.s006.docx]

Dear Editor

 Thank you for considering our revised manuscript. Here are our responses to your comments and our changes according to your and the reviewers’ suggestions.

Response to comments

Editors comments:

*The manuscript still needs to be heavily edited for English grammar, etc, and the typos in* ***Figures fixed as noted by the reviewers.***

***“Positional cloning” is an inexact term*** *that only partially describes your efforts.*  ***You used high resolution mapping to narrow a candidate genomic region.  This was followed by sequence analysis to identify a most probable SNP in Clec4b, followed by functional studies to test that contention.***

*As noted in the reviews, the evidential support that Clec4b is responsible for the phenotypes is indirect without complementation or some other direct test.  We suggest that you* ***temper the interpretations and conclusions in that regard****. What you have accomplished is to* ***provide a several lines of indirect support*** *for your contention, importantly including new data with the anti-Clec4b antibody (Fig 3F).*

**Response:** We have now edited the manuscript for English grammar and typos.

We have now changed the title which now reads: Identification of *Clec4b* as a novel regulator of bystander activation of auto-reactive T cells and autoimmune disease.

We have now modified the main conclusion regarding the link of DCAR polymorphism to some of its functional effects to be based on several type of data but not proven by two different conclusive complementary experiments. In the result section 1 regarding the genetic mapping conclusions have now been rephrased to: ” The inherent ability to reproduce linkage in an F2 cross between DA and BN provided sufficient evidence to us to conclude that arthritis regulation most probably stems from variations in *Clec4b* rather than *Clec4e*. We identified a causative nonsense SNP in the *Clec4b* DA allele that causes a truncation of the DA allelic protein that is nonfunctional but with expressed transcripts. This will make the DA strain to be a natural knock-out for Clec4b, which further substantiates the gene as the arthritis regulating gene in the APLEC region.”

**Reviewer #1:**

1. *The authors addressed some of my concerns by providing additional data supporting the view that Clec4b contributes to autoimmunity. However, a complementation assay was not performed.*

**Response:** We assume that the suggested complementation assay would be to make a corresponding targeted mutation but this has not been possible with respect of the timing of the project. Initial experiments have been done with siRNA silencing of Clec4b but each in vitro experiment has either resulted in low efficiency in transfection-rate or low viability of the myeloid cell population. However, we are making a Clec4b2 knock-out mouse that have been derived to viable germ-line cells and mice will soon be available but will not reach our animal house yet for half a year. Which will set us back the project for years, since the Clec4b strain need arthritis predisposed MHC. Nevertheless, the data are based on considerable amount of work over the years as it needed a series of recombination and testing of new congenic strains to minimize the fragment in completely hypothesis free approach. The minimal fragment distinguishing a few mutations is too small to split in reasonable time frame, but we think we have provided several lines of experiments to prove that the polymorphism in DCAR is a causative genetic effect. It does not exclude other linked mutations conclusively, but this is not the goal. We have also provided functional evidence on how the DCAR polymorphism could mediate the effect on arthritis. We have now tried to clarify this level of experimental evidence and hope to have concluded it in a modest way.

*2. Some of the text still needs revision. This needs to be corrected by an English-speaking editor. It is so difficult to read at times that I am not sure I understand the experimental approach. Here are some examples (please don't limit your corrections to these few examples).*

**Response:** An English-speaking editor has now helped to correct the language. We hope it is now satisfactorily improved. It also included the specific points raised:

*Therefor (needs an e)*

**Response:** This typo has been corrected in the author summary and throughout the text.

*"****When developing tissues specificity****"* ***This is in the intro****. It is not clear what is meant here.*

**Response:** The full sentence has been replaced by “Innate immune cells interpret danger from a range of pattern-recognizing receptors (PRRs) on their cell surfaces. when the innate cells sense enhanced risk in the environment, these cells are able to activate other cells, such as T cells. If the activation of adaptive responses displays joint specificity, the situation could initiate clinical arthritis. “

*A few sentences start with "****Suggesting****". This needs to be corrected.*
**Response:** In one instance at page 14 the Suggesting word is replaced by “this suggests”. The sentence on page 7 has been altered to the following “The inherent ability to reproduce linkage in an F2 cross between DA and BN provided sufficient evidence to us to conclude that that arthritis regulation most probably stems from variations in *Clec4b* rather than *Clec4e*.”

***Supernatant usually does not contain cells.*** *Do the authors mean "flow through"?*

**Response:** Supernatant has been replaced by “flow—through” throughout the manuscript.

*effects vs affects*

**Response:** This has been corrected by an English speaking editor and only proper usage of the two words through-out the text.

*A sentence starts describing figure 6 with "****Most likely****" - this sentence structure needs to be revised*.

**Response:** This sentence has been changed to ”The CD4^+^DC mediated T regulation occurs, most likely, by limiting bystander activation via cytokine secretion.”

*Capital letters are used inappropriately in words in mid-sentence.
etc…*

**Response:** Capital lettering is now not used inappropriately in the text.

**Reviewer #3:**

*The manuscript entitled "Positional cloning of Clec4b - a novel regulator of bystander activation of auto-reactive T cells and autoimmune disease" proposed that Clec4b is a novel regulator expressed in CD4+ DCs and limit T cell activation. Although the authors did not clearly address the molecular mechanism mediated by Clec4b,* ***the identification of candidate polymorphism by positional cloning is significant.*** *To confirm the role of Clec4b directly through genetical approaches, the authors may want* ***to perform CRISPR/Cas9-mediated complementation in DA rats and/or introduction of the same mutation in E3 rats.***

**Response:** Please see our answer to reviewer one.

***Have all data underlying the figures and results presented in the manuscript been provided?*** *2. Reviewer #3: No:* ***The mRNA expression of each Clec would be better to be shown as ∆∆Ct.***

**Response:** We have kept the 2***∆∆Ct*** since the primers for each *Aplec* encoded gene were chosen from 4 different primer sets merely because of their amplification-efficiency being between 95% to 105%. This to ensure that we can use the logarithm base of 2.
