# Supplementary material for: Identification of Clec4b as a novel regulator of bystander activation of auto-reactive T cells and autoimmune disease
Source: PLoS Genet. 2020 Jun 4;16(6):e1008788. doi: 10.1371/journal.pgen.1008788 (PMC7297379; doi:10.1371/journal.pgen.1008788)
Supplement: Attachment — Submitted filename: plosgen response 200405.docx [file pgen.1008788.s007.docx]

Dear Editor, PLOS Genetics,

We have now revised the paper 'Identification of Clec4b as a novel regulator of bystander activation of auto-reactive T cells and autoimmune disease'. We have made corrections following all the suggestions and also rewritten it slightly to update the English grammar.

We are happy to make additional corrections if needed.

We remain for your decision.

Sincerely

Rikard Holmdahl

Point to point reply:

While we continue to believe that this study deserves communication by PLOSgenetics, consistent with the reviewer’s previous comments, we believe that definitive causality of the Clec4b missense SNP continues to be overstated in the revised manuscript.

1. In Abstract: “We have positionally cloned a non-sense loss of function single nucleotide polymorphism in the C-type lectin receptor, Clec4b, and have shown that it controls chronic autoimmune arthritis in rat models of rheumatoid arthritis. “

Authors: We suggest changing to “We have identified a non-sense loss of function single nucleotide polymorphism in the C-type lectin receptor, Clec4b, and have shown that it controls chronic autoimmune arthritis in rat models of rheumatoid arthritis. “

2. In Introduction: “We have now located the causative single nucleotide polymorphism (SNP) in the Pia7 locus where it functions in regulating the Clec4b gene.”

**Authors: We suggest changing to “We have now located a single nucleotide polymorphism (SNP) in the Pia7 locus, regulating the Clec4b gene.”**

3. In Discussion: (1st para) “The identification of a single nucleotide polymorphism controlling the expression of Clec4b/Dcar in the rat has been found to be of major importance for the regulation of autoreactive T cell activation and the development of arthritis.”

**Author: We suggest changing to: The identification of a single nucleotide polymorphism controlling the expression of *Clec4b* in the rat highlights a new role of *Clec4b* in regulation of autoreactive T cell activation.**

4. The 1^st^ paragraph of the discussion would be a good place to rationalize why the you consider the SNP to be the likely cause of the QTL.

**Authors: We have now rephrased the 1^st^ paragraph of the discussion into “The identification of a single nucleotide polymorphism controlling the expression of *Clec4b* in the rat highlights a new role of *Clec4b* in regulation of autoreactive T cell activation. The suggested identification of the *Clec4b* SNP to be causative was based different type of evidence. Firstly, it was found within a 200kb chromosomal interval defining the QTL Pia7. This genomic region harbors three protein coding genes, whereof two had non-synonymous SNPs. Of these, only the SNP in *Clec4b* showed functional evidence as it led to differential gene expression in naïve rats. Secondly, data from several genetic mapping studies aiming to identify arthritis susceptibility regulating loci in the rat, shows that only the DA version of Clec4b segregates with disease susceptibility, and not strains lacking the *Clec4b* SNP but maintaining the polymorphism outside the *Clec4b* locus. Thirdly, only the DA *Clec4b.E3* congenic, and not the DA rat, expresses the Clec4b protein. More specifically, Clec4b is highly expressed on CD4+ DCs, a population known to be regulating MHCII interaction and T cell activation. Interestingly after an intradermal oil injection, the DA strain displays a rapid onset of autoreactive T cells whereas *Clec4b.E3* rats have normal control of T cell activation after oil injection. *Clec4b* is a C-type lectin receptor, expressed in classical CD4^+^DCs, and we now find that it is essential for the control of bystander activation of autoreactive T cells during immune priming. Deficient expression of *Clec4b* allows the escape and expansion of autoreactive T cells leading to arthritis.”.**

In addition, we would like to add that we have edited the manuscript for its grammar.

We have now uploaded a striking image with the following caption.

**“A rat cross with segregating genes. This study is based on classical genetic mapping techniques to identify the arthritis susceptibility genes in the DA rat.”**
